# AtAwAR Translate: Attention-Aware Language Translation Application in Augmented Reality for Mobile Phones

**DOI:** 10.3390/s22166160

**Published:** 2022-08-17

**Authors:** Lisa-Marie Vortmann, Pascal Weidenbach, Felix Putze

**Affiliations:** Cognitive Systems Lab, University of Bremen, 28359 Bremen, Germany

**Keywords:** translation, augmented reality, brain–computer interface, EEG, smartphone, attention

## Abstract

As lightweight, low-cost EEG headsets emerge, the feasibility of consumer-oriented brain–computer interfaces (BCI) increases. The combination of portable smartphones and easy-to-use EEG dry electrode headbands offers intriguing new applications and methods of human–computer interaction. In previous research, augmented reality (AR) scenarios have been identified to profit from additional user state information—such as that provided by a BCI. In this work, we implemented a system that integrates user attentional state awareness into a smartphone application for an AR written language translator. The attentional state of the user is classified in terms of internally and externally directed attention by using the Muse 2 electroencephalography headband with four frontal electrodes. The classification results are used to adapt the behavior of the translation app, which uses the smartphone’s camera to display translated text as augmented reality elements. We present the first mobile BCI system that uses a smartphone and a low-cost EEG device with few electrodes to provide attention awareness to an AR application. Our case study with 12 participants did not fully support the assumption that the BCI improves usability. However, we are able to show that the classification accuracy and ease of setup are promising paths toward mobile consumer-oriented BCI usage. For future studies, other use cases, applications, and adaptations will be tested for this setup to explore the usability.

## 1. Introduction

In our globalized age, many aspects of our lives, such as travel, advertising, business collaborations, signage, and information texts, are becoming increasingly international. Languages, however, remain very national and, along with culture, are often one of the most obvious differences between countries. They pose a big communication issue.

Encountering material in a foreign language, for example when traveling, makes information intake difficult and requires the use of a translator. Although dictionaries were commonly used for such purposes in the past, translators on cell phones are now far more widespread in the digital age. In some circumstances, translating single words or sentences is sufficient; in others, entire documents must be translated into the native tongue. Copying such texts into a cell phone is time-consuming, and it becomes especially tough when the foreign characters differ greatly from your own. One solution to this problem is the camera-based translator, which can recognize the foreign language text and display translations by using augmented reality (AR) [1]. Such AR translators can be used to swiftly construct a translated version of what the user is seeing. In real-time, text in an image is identified, translated, and replaced with visually corresponding translations. This produces an almost instantaneous AR illusion. It excludes the need for text to be typed and highly decreases the time required to obtain a translation. On the downside, this continuous updating of potentially salient visual content increases the chance for distraction from thought processes [2].

In this work, we explore how mobile AR translators for smartphones can be improved by adding attention-awareness. The attentional state of a user is estimated by using an electroencephalography-based (EEG) brain–computer interface (BCI) in which the behavior of the application is adapted to the current state. It has been shown in several studies that an efficient use of AR can have positive effects on mental workload and task performance unless the distraction level of the virtual content is too high [3]. The virtual text overlay on the screen is usually updated regularly when using AR translators. This is required to compensate for minor unintentional camera movements and to update the translation in real time if new words are revealed. These updates can be a source of distraction, especially during times of internally directed attention (i.e., thought, mental task solving, memory) [4]. When users direct their attention internally, the suggested system reacts by halting the translations, which would otherwise update continually.

Recent machine learning research showed that it is possible to separate attention into internal and external attention with decent accuracy [5]. However, the applicability of this technology has mainly been demonstrated with BCIs that rely on a more or less stationary EEG setup, severely limiting the mobility of its users. Several companies have recently produced consumer-oriented EEG headsets that emphasize comfort and mobility. This technology represents a significant step toward the widespread application of BCIs. We considered an AR translator an appropriate use-case scenario to demonstrate the enhancement of AR applications by adding attention awareness with a mobile BCI because it is commonly used “on the road”. Previous research has shown that the adaptation of an AR smart-home system based on internally or externally directed attention decreases the distraction and increases the usability compared to an attention-unaware system [6]. However, the study was performed inside a lab by using a head-mounted AR device and an EEG cap with 16 electrodes that is meant for research purposes only. We will focus on a consumer-oriented BCI that is cheaper, has an easier setup and calibration, and can easily be used "in the wild" to improve the smartphone application.

The two main goals of this work were the implementation of the cell phone application in combination with the mobile BCI and a user study to evaluate our hypotheses about the application’s improvement when attention sensitivity is incorporated. The main contribution of this work is the purely smartphone-based setup using a lightweight consumer-grade EEG headset as the BCI data source. To test the system, we built the AR translator following the current-state-of-the-art apps. The final application is an attention-aware AR translation app that we accordingly named “AtAwAR Translate”. The adaptability to the user’s attentional state was included as an optional parameter to compare two versions of the system—the attention-aware one and the standard one—in the user study. A travel-themed scenario was used for this study, in which participants had to read and understand foreign posters before answering questions on the material. The evaluations of the system will be based on achieved classification accuracies and user questionnaires rating the system’s usability and level of distraction. The results will also provide insights regarding a design that accounts for false classifications. Assessing the potential benefits with the potential drawbacks and finding the right balance on when to adapt is key to maximizing the improvement.

## 2. Related Work

Studies related to this work cover other AR translation applications, internal and external attention classification from EEG data, and mobile BCI setups. We are not aware of previous works that implemented and tested a mobile, lightweight BCI setup for cell phone applications that differentiates attentional states of the user. The combination of biosignal data recordings and augmented reality has recently been of high interest, for instance, to explore neurocognitive processes in real-world environments without losing experimental control [7]. Zhao et al. [8] designed an AR-based mobile photography app that detects emotional states by using EEG data to improve learning about photography and to decrease the cognitive load while achieving a better emotional state. Following a similar goal of reducing the cognitive load, Yan et al. [9] used eye tracking data in an AR parcel scanning task to integrate foveated vision detection and smooth pursuit of eye tracking. Their results also showed facilitated task solving in AR.

### 2.1. AR Translation

Text can be translated from and to a language of choice by using a translator, typically by inputting text manually. An AR translator directly projects translations onto existing text in the actual environment with the help of a display. The most well-known example is the Google Translate App [1]. There is no need for human text input because the translator detects text automatically. An AR translator must detect and recognize text, which is accomplished by optical character recognition (OCR). Following that, the text is translated and projected back into position onto the original text, preferably with a text style that resembles the original for authenticity. To produce an authentic illusion of existence, the translated text must not only be applied in the precise location, but must also move with the camera and underlying text. This is accomplished by object tracking. The exact implementation of the application will be described in a later section.

The number of research publications on AR translators is limited. One of the latest papers concerning AR translators is from 2017 in which Tatwany and Ouertani [10] review AR use for text translation. They identified 12 papers relevant for their research. Most papers present translators that work by taking a picture. The picture is then analyzed with OCR and text is overlaid onto it, usually in a different location in a different style. Because translations are displayed on a fixed image, these translators cannot be considered true AR translators because they do not work in real time.

Among all papers, only the TranslatAR (2011) [11] used real-time translations. Users translate text by tapping onto text, and translated versions with matching styles emerge at the same location after roughly one second. The translated text then moves in sync with the camera or the text, as if it were glued to the original text (text stickers). Using TranslatAR, text stickers move with the actual text, based on tracking. While Head-mounted display-based translators exist [12], smartphones as handheld AR devices dominate the research [10].

The latest publications on AR translators were in 2015 [10,13], thus, it is interesting to see how consumer products evolved since. In 2022, numerous consumer translation apps were available, several of which allow for language translation by using the smartphone camera (see Table 1 for top Google Play Store results). The majority of the apps evaluated provided this functionality, but only the top four of these apps were included in the table. Only three apps were found to have AR capabilities, in which text is displayed within the real-world surroundings. Two of them in particular overlay the translation on the text and return an image in which the original text can still be seen behind the translations. Because translations are displayed as images, these two translators are not real time and hence do not fully meet the AR standards. Google Translate [1] is the only app that features real-time translations and is therefore the only true AR translation app. It is also the only consumer app with genuine text stickers. Although TranslatAR [11] has this capability, it only translates and tracks a single word at a time, whereas Google Translate replaces all words. Google’s AR translator also allows one to manually pause translations, effectively freezing the screen. Google Translate was launched in 2010, but it was not until 2015 that it featured AR translation. Word Lens was the most popular AR translation app at the time and was a primary reference for most AR translation-related research [10]. Google Translate integrated Word Lens into their application in 2015, becoming the market’s leading AR translation software with over 500 million downloads to date. According to download statistics, Google Translate has a significant market dominance, with ten times more downloads than its closest competitor, Microsoft Translate. The Google app is also the first translation app to appear in the Apple App Store when searching for “translator”.

Google’s AR translator is certainly the most advanced and cutting-edge, and it will be used as a model for this work.

### 2.2. Attention Classification

Attentional mechanisms are applied to filter task-relevant from task-irrelevant sensory input and mental processes at all times to deal with the constant information overload. Thus, attention can be described as the mental process of concentrating on certain perceivable information. The processes, layers, and dimensions of attention are numerous. One distinguishable aspect of attentional mechanisms are internally and externally directed attention [5]. In times of internal attention, sensory input is rated as task-irrelevant and the focus is on internally produced information (i.e., thoughts, memories, mental problem solving). External attention, on the other hand, is a selection and emphasis on information offered by our environment [4]. For the suggested use case, reading the translations required external attention and thinking about the question/task requires internal attention. In this study, we will interpret neurophysiological activity recorded by an EEG to produce a quantitative assessment of the given attentional state. Other studies instead use eye tracking data as classification input [14,15,16].

Cooper et al. [17] found that alpha band amplitudes are higher during periods of internal attention, which they attribute to active blocking of external input. According to Benedek et al. [18], right-parietal alpha power increases with internal focus. Putze et al. [19] were the first to distinguish between internal and external attention using an EEG and linear discriminant analysis (LDA) on a single trial, achieving an accuracy of up to 81.2%. Vortmann et al. [14] employed a multimodal setup to categorize internal and external attention in a real-time evaluation, employing both EEG and eye tracking, with an accuracy of 60.9%. In another study, Vortmann and Putze [6] improved the usability of a smart-home system with the multi-modal setup of the previous study, reaching a real-time accuracy of 65.7%.

As previously stated, we will use a lightweight, low-cost EEG headband for the BCI in this study in order to improve the usability and application possibilities of the proposed system. We will work with the Muse 2 Headband by InteraXon Inc., Toronto, Canada [20]. The number of electrodes in the Muse Headset is less than that in the aforementioned research. However, the right parietal area is partially covered by the TP10 electrode, which appears to be a significant area for separating attention into internal and external, as previously indicated. This gives reason to believe that attentional classification into internal and external attention is achievable, despite the fact that electrode coverage is restricted and data quality is worse. Previously, consumer-grade EEG was used to determine attention in passive BCI scenarios; however, classifications referred to attention levels in terms of focus and involvement [21,22,23,24]. To the best of our knowledge, using a consumer-EEG, such as the Muse Headset, to classify internal and external attention was not done before. We will use the predicted attentional state of the EEG data classification to adapt the behavior of the AR translation application.

### 2.3. Mobile and Passive BCIs

Brain–computer interfaces allow for direct communication between the brain and an external device. Mobile BCIs eliminate the need to stay stationary that traditional BCIs have due to their wiring. As BCI technology becomes more popular, several businesses are working to develop mobile, pleasant, and non-invasive EEG solutions. Because of its setup and method of data collection, EEG counts as a non-invasive brain activity recording modality and is frequently used for BCIs [25]. It is a reasonably cheap and effective recording technology [26]; hence, EEG is one of the most popular types of BCI, having been employed in 60% of BCI research from 2007 to 2011 [27]. However, laboratory EEG necessitates a time-consuming setup and cleaning procedure [28]. A trained specialist is required to place the electrodes correctly [29], and to apply the conductive gel or saline solution that is routinely used to promote scalp connection, which improves data quality [28]. A mobile BCI, on the other hand, increases usability and aesthetics by eliminating the need for lengthy electrode placement procedures. It can also be used dry, avoiding the need for the time-consuming application and subsequent removal of conductive gel. This, however, comes at the expense of data quality. As previously stated, research EEG needs the placement of a qualified professional, whereas mobile EEG can be placed by a novice. Consumer-oriented BCI solutions, such as headsets or headbands, are offered by companies such as “Emotiv (EPOC), Neurosky, Advanced Brain Monitoring (B-Alert X10), InteraXon (Muse), and Melon” [28]. The Muse headset was validated for EEG research by Krigolson et al. [30].

Active BCIs are used to consciously control a system by deliberately changing one’s thoughts to evoke a certain action of the system. Zgallai et al. [31], for instance, used the Emotive mobile EEG headset to control a smart wheelchair with four different movements. For a passive BCI, users do not need and should not alter their way of thinking actively [32]. It is meant as an implicit interaction in which the passive BCI picks up automatic, spontaneous brain activity for a background monitoring of cognitive and affective states. The implicity is defining for passive BCIs; the user should utilize the system as if there was no passive BCI [26].

Passive BCIs have been utilized frequently in research over the last few decades, although largely in laboratories or tightly controlled situations [33]. Recently, research on applications in real-world settings is emerging. In Roy and Frey [34], passive BCIs help users under substantial stress and cognitive load, such as air traffic control or drone management. They accomplish this by modifying the information presented on an interface in order to reduce task complexity and hence workload. A passive BCI can also be used to update the user interface depending on user-experienced problems [32]. Zander et al. [35] assessed the use of a passive and mobile EEG (actiCAP Xpress dry-electrode) for autonomous driving in terms of signal quality and usability and discovered that the prerequisites for the development of actual systems were met. An autonomous automobile might utilize the data to determine if the driver is ready to take over control, or to assess the user’s mood or attentional state.

In this work, we will make use of a passive mobile BCI together with a smartphone application aiming at an easy, fast setup and effortless usability to decrease the distraction caused by AR applications.

## 3. The Mobile BCI-Smartphone System and Application

The AR translator implemented for this work is a replication of the Google Translate app, because it proved to be the most advanced and state of the art. We implement our own version of the app to be able to make the necessary changes for the BCI integration and because the source code is not publicly available. Translated written text is presented dynamically in real-time by using text stickers that create a believable AR experience. When users read and process a translated text, they alternate between internal (no sensory input required) and external (focusing on perceptual input) attention. During internal attention, perceptual input is usually suppressed to a certain degree. However, the possibly distracting and unnecessary updating of the visual text stickers is a very salient change that would disrupt internally directed focused attention. Therefore, pausing the updating of the visual augmented content while users are internally focused is an excellent use-case to test a passive BCI for attention sensitivity. We will determine the attentional state and modify the interface’s behavior accordingly. The update frequency, appearance of text stickers, location of text stickers, and changes in appearance and location between updates determine the level of distraction. Our application can suspend the process of updating text stickers when the attention is internally focused and as soon as the user turns their attention externally, the updating of text stickers can resume. We will use a Muse 2 Headset to avoid limiting the mobility of users and for a fast and easy setup.

### 3.1. Translation App Implementation

We had to prioritize rapid computation and execution times when developing the AtAwAR Tranlate system as a real-time AR translator. Balancing performance and efficiency was key when selecting and developing algorithms. The main app was written by using Java and C++. The app operates by using an image resolution of 540 × 960 pixels, which was found to be the optimal compromise between performance and quality. (The images of the app presented in the following were taken by using a higher resolution (720 × 1280)). The essential features of the AR translator are provided in the following sections to provide a brief structural overview.

**Figure 1:**
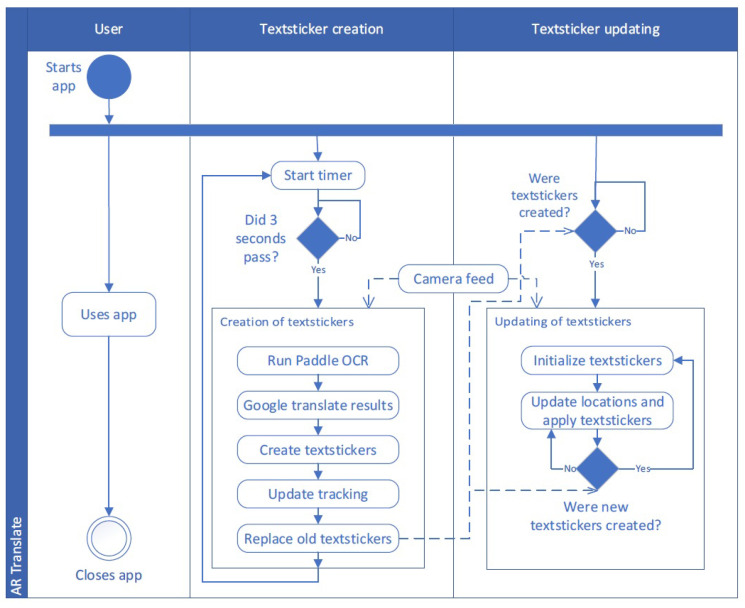
Activity diagram for the implemented AR translator showing the user activity, the creation of text stickers, and the updating of the text stickers. The attentional state of the user was is not supplied yet.

The activity diagram in Figure 1 visualizes the structure of the AR translator. After the user launches the app, the main processes are started.

#### 3.1.1. Text Sticker Creation

First, the text-sticker production process begins with a timer, which serves two purposes: as a delay for the camera to establish focus and as a wait between updates for the duration of the app’s existence. The time it takes to create new text stickers ranges between 300 and 3000 ms. It is expected that enough time has passed for the text stickers to be changed after this amount of time. The open source PaddleOCR, which is neural network-based and under active development with frequent releases, was used in this work for optical character recognition (OCR) [36]. The ultra-lightweight OCR is fast and has a high accuracy [37], even on scene text, making it ideal for this application. To generate the texts, an image is sent into PaddleOCR’s model, which does text detection, recognition, and classification.

Following that, the discovered labels are forwarded to Google’s translation service through REST API. The translated labels and the original image are combined to create text stickers with the same text and background color, font size, and thickness as the translated text. For each label detected by OCR, a region containing the label’s location in the image is returned. This is accurate, however, because OCR and text-sticker production take time, the text may have changed in the meantime, invalidating the previously accurate detected areas. As a result, the identified text regions are updated after the generation of the text stickers to reflect reality. After text sticker creation is complete, the image utilized for the OCR model is compared to the initial picture. By using feature matching, the label regions from the OCR image are redetermined in the latter picture [38]. This method significantly reduces the latency to reality, allowing optical flow to be exploited for real-time tracking. Optical flow attempts, at best, to determine where points from one image went to in another similar image [38]. Following that, any existing text stickers are replaced with the text stickers generated by this update.

#### 3.1.2. Text Sticker Update

The updating of text stickers is accomplished by the use of optical flow. It begins by locating four tracking points within a label region for each text sticker. These tracking points can be identified again in a new image, and the placements and shapes of the text stickers are modified based on the difference. To help overcome the latency caused by feature matching, a wider search window size is used for the initial application of optical flow after feature matching. Following that, a reduced window size is used to boost speed, allowing for a higher frame rate. This is repeated frame after frame until the text stickers are changed with updated ones.

**Figure 2:**
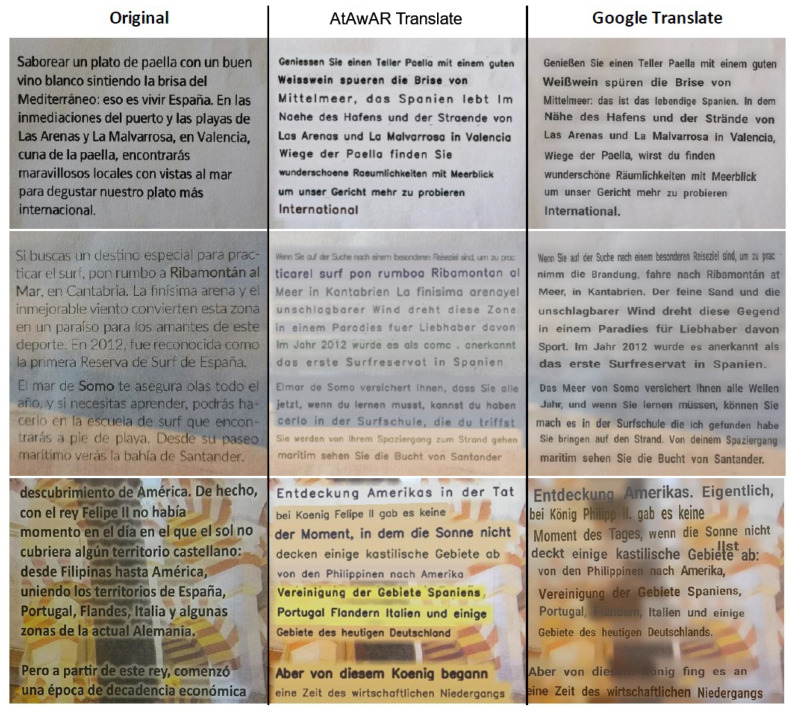
Comparison of the implemented AR translator (AtAwAR Translate), Google Translate and the original text for three different text and background combinations.

Figure 2 shows examples of original text, our AR translation application and the Google translate results.

### 3.2. Brain–Computer Interface

In the following, the addition of the BCI-based adaptive pausing is described. This includes how the data from the Muse Headset is transferred to the app, and how that data is processed and classified into either external or internal attention.

**Figure 3:**
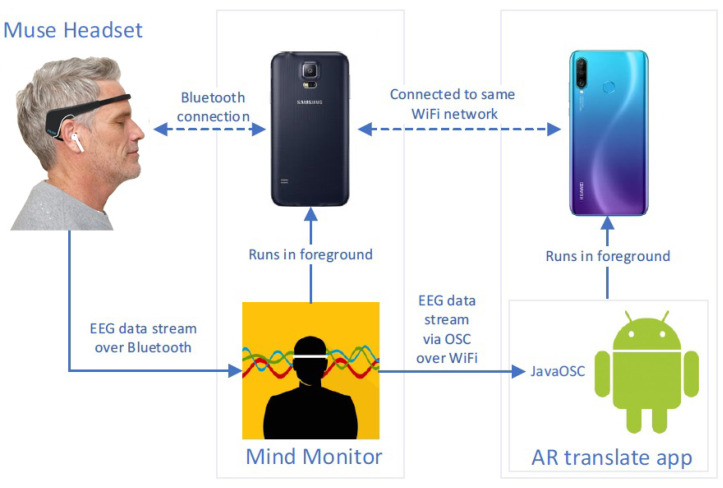
Schematic description of the data flow of the implemented system. The Muse 2 headset is connected to a fist smartphone via Bluetooth and sends the EEG data stream to the installed Mind Monitor app. A second smartphone that is connected to the same WiFi network is running the AR Translation application and receives the EEG data via OSC over WiFi from the first smartphone.

Figure 3 gives an overview of the data flow structure from the Muse Headset to the app, which will be described in the following. The data is first sent from the Muse Headset to an app called Mind Monitor [39]. Once linked to the Muse Headset, the app displays several graphical representations of the EEG frequency spectra as well as an indicator of the headset’s fit, which is useful for determining whether the data from Muse is being captured adequately and the device is being worn correctly. Furthermore, Mind Monitor applies a 50-Hz notch filter to remove power line noise.

Mind Monitor streams the data via Open Sound Control (OSC) by using the user datagram protocol (UDP), acting as an interface to the Muse Headset’s data. The OSC data is streamed to a device via WiFi by specifying the receiver’s IP address. Mind Monitor is running from a separate device for the following reasons.

- To reduce interference from the OS Android (the Mind Monitor should run in foreground);
- Because the AR translate app is already computationally demanding, it makes sense to not put any extra strain on the device;
- Because it enables continuous monitoring during the execution of the study, to ensure that there are no issues with the connection between Muse and Mind Monitor.
- Because the creator of Mind Monitor mentioned that the Muse Headset has connection issues with the Bluetooth module of Huawei phones, which was used for the translation app devices.

To receive the OSC data from Mind Monitor, the library JavaOSC [40] is used. When OSC streaming is enabled in the app, the Mind Monitor app provides multiple data streams over OSC pathways. The subscribed items are as follows:

- **EEG** absolute values of delta, theta, alpha, beta, and gamma frequency bands as four float values (frequency ranges: δ(1–4 Hz), θ (4–8 Hz), α (7.5–13 Hz), β (13–30 Hz), γ (30–44 Hz);)
- **Horseshoe** indicating fit of the electrodes;
- **Battery info**;
- **Gyroscope** measuring or maintaining orientation and angular velocity;
- **Accelerometer** measuring acceleration;
- **Blink**; and
- **Jaw Clench**.

The absolute power band frequencies will be used for the attention classification process.

The Muse Headset can be seen in Figure 3. It is a four-channel EEG with two silver sensors on the forehead and two conductive silicone-rubber sensors on the ears. It can communicate data wirelessly via Bluetooth 4.0 and samples data at 256 Hz. The electrodes cover areas of the brain in the anterior frontal (AF7, AF8) and temporoparietal (TP9, TP10) lobes. It also has a 3-axis accelerometer and gyrometer for tracking head motions [41].

The Muse Headset is adjustable in length and is thus suits a wide range of head sizes. It is a low-cost EEG designed to be used as a personal meditation helper. With a price of $250, it is far less expensive than research EEG devices (e.g., ActiChamp 75,000 $). In terms of data validity, Krigolson et al. [30] assessed the Muse data of 1000 participants in varied contexts and demonstrated its robustness and accuracy in a visual oddball task. The Muse Headset’s detection patterns were found to be identical to those of the research-grade wet EEG systems actiCHamp and g.Tec [42]. In noisy environments, Przegalinska et al. [43] critique low data quality. Additional Muse Headset problems were discussed in those studies: Bluetooth-related delay and jitter (20–40 ms delay, jitter 5 ms), a temporal unstable beginning of consecutive samples (time difference of −10 ms to 150 ms), and missing samples (0.01–0.05% missing samples across all participants).

#### 3.2.1. Classification

The adaptation of the AR translation application behavior was based on the classified attentional state. We differentiated internally and externally directed attention based on the recorded EEG data. Following the results of Putze et al. [19] and Vortmann and Putze [44], we chose 4-second data windows for the feature extraction of single trials. The classification was performed person-dependently, and 400 s of training data were collected per person to train an LDA. The training phase of the classifier will be explained in more detail in the user study. The extracted features were provided by the MuseIO [45]. To calculate the absolute band powers of the aforementioned frequency bands, a Fast Fourier Transform is used on a 256 sample Hamming window, sliding at 10 Hz. For each electrode and frequency band, one sample is extracted every 100 ms for the previous second. Thus, a total of 20 features is contained in the feature set and the windows overlap with 90%. As 400 s of training data were recorded per participant, a total of 4000 feature vectors were available per participant. The LDA was implemented by using the standard scikit-learn parameters and settings because they lead to good results on pilot data and hyperparameter optimization per participant would be time inefficient and reduce the usability of this online classifier. To calculate the training accuracy for each participant that was used to evaluate the quality of the calibration and training phase, a 5-fold cross-validation was performed on the data, and the average was calculated over all folds. The results will be reported later. For the final training of the LDA, all feature vectors of available training data were used.

Apart from the notch filter, no additional preprocessing of the data was performed to keep the computation times at a minimum.

#### 3.2.2. Prediction Integration

In the following, the strategy to adapt the BCI behavior based on the classification results of the BCI data will be explained.

We expect that while reading something that is relevant for a task or question, the user switches between internally and externally directed attention. During the externally directed attention the user is sensitive to perceptual input and scans or reads the available information. During internally directed attention on the other hand, the user is focusing on internally generated information, such as thoughts or memories. For instance, to integrate the newly acquired knowledge from the external input into existing knowledge or to formulate an answer to a question. The perception of external stimuli is suppressed but salient changes of external stimuli, such as the smartphone display in our system, can be distracting. An update of the displayed content or translations could be such a distracting change. Thus, whenever the attentional state of the user is presumably “internal”, the updating of text stickers is paused. However, the pausing of translation updates in times of externally directed attention is presumably not diverging the attention from the current goal and therefore the text stickers can be updated whenever necessary.

The pre-trained classifier predicts the attentional state every 100 ms based on the last second. We compile the predicted labels of 4 s to make a decision whether to pause the translation updates or not. Within the 4 s, at least 60% of the predictions (n = 40) have to be labeled “internal” for the translations to pause. This threshold was chosen to create a bias toward “not pausing”, as wrongful pausing during external attention was considered more impairing, whereas missing pausing during internal attention can be tolerated and is typical in attention-unaware systems.

## 4. User Study

In a user study, the AtAwAR Translate system was tested with and without the addition of attention-based pauses. This gave information about the performance and usefulness of the AR translator, as well as the modality preferences of the participants. Furthermore, the modality with BCI was examined in terms of attentional classification accuracy and distribution, as well as the length and correctness of pauses.

The study’s hypotheses are stated first, followed by the methodology, which explains how the study was carried out. Following that, the results are displayed and discussed in the last section, highlighting potential causes of faults and recommendations to enhance the system.

### 4.1. Hypotheses

One of the goals of this research was to evaluate the usability of a mobile BCI for attention awareness. Several hypotheses concerning this purpose were considered.

#### 4.1.1. Main Hypotheses

The main hypotheses relate to the positive (*H1*) and negative (*H2*) effects that the pausing of translation updates may cause. It is believed that although users have internal focused attention, the pausing of translations is perceived as positive (*H1*). Contrary to that, pauses during phases of external attention, which occur during reading, are believed to have a negative effect (*H2*).

**Hypothesis** **1.** *The larger the percentage of pauses during thinking, the higher the experienced usability and the lower the task load.*

**Hypothesis** **2.** *The larger the percentage of pauses during reading, the lower the experienced usability and the higher the task load.*

As the app makes decisions for the user that are not always correct, attempting to help the user by pausing when they are thinking inevitably comes with negative effects while reading. *H1* and *H2* with regard to the attentional prediction accuracy gives insights to how high the accuracy needs to be to improve the app, as the accuracy is a deciding factor for how the usability regarding the pausing is perceived by participants.

#### 4.1.2. Other Hypotheses

The other hypotheses relate to whether text appearance or content may influence the experienced usefulness of the pausing.

**Hypothesis** **3.** *The more distracting the displayed translation results are, the more helpful pauses are during thinking.*

**Hypothesis** **4.** *The more demanding the combination of text and question is, the more helpful pauses are during thinking.*

Answering these hypotheses shows for which types of text the pausing is most useful. Another key goal of the study is to test the AR translator, for which it makes sense to test various texts that differ in visual and contentual complexity, ergo *H3* and *H4* can be tested with little extra effort. Another purpose of the study, as previously stated, is to evaluate the AR translator. This comprises not only the operating principle of the AR translator, but also the two modalities of pausing and not pausing. This assists in determining where the AR translator can be enhanced the most and where it already performs adequately. Evaluating the two modalities in relation reveals whether pausing is advantageous or not, and why.

### 4.2. Methods

The research was conducted by following a within-subject design. Each participant tested the app with and without the Muse Headset. The evaluation of the hypotheses were mainly based on questionnaire results. To test the translation app, eight posters with Spanish texts were created. For each participant four posters were used for the version with Muse Headset and four for the version without. The four permutations of these combinations were labeled modalities A, B, C, and D, which were rotated throughout the study’s runs. The participant count was multiple of four. Thus, the poster distribution across both versions was balanced.

#### 4.2.1. Participants

Fourteen healthy participants between 16 and 59 (mean age 27.1, SD 9.8) with normal or corrected eyesight who were all German native speakers participated in the study. Two participants were excluded because the attention prediction was excessively unbalanced, making the BCI version unsuitable (nearly always paused) or too similar to the BCI version (almost no pauses). This reflects the expected rate of people with BCI illiteracy (the inability to control a BCI accurately) [46]. To keep the equal distribution of poster combinations, two new volunteers were selected for the excluded participants’ experiment versions. The volunteers did not receive monetary compensation. Eight of the remaining participants were male and four female. The majority of participants did not speak Spanish at all, and those who did had just a rudimentary understanding. Two of the participants had previously used an AR translation software (both Google Translate). Each participant was informed about the collection of EEG data and written participation consent was collected. All participants were recorded in the same room that was not specifically shielded to test the system under non-laboratory conditions. The data was anonymized by assigning six-digit participant IDs at random and the study was approved by the local ethics committee.

#### 4.2.2. Procedure

**Figure 4:**
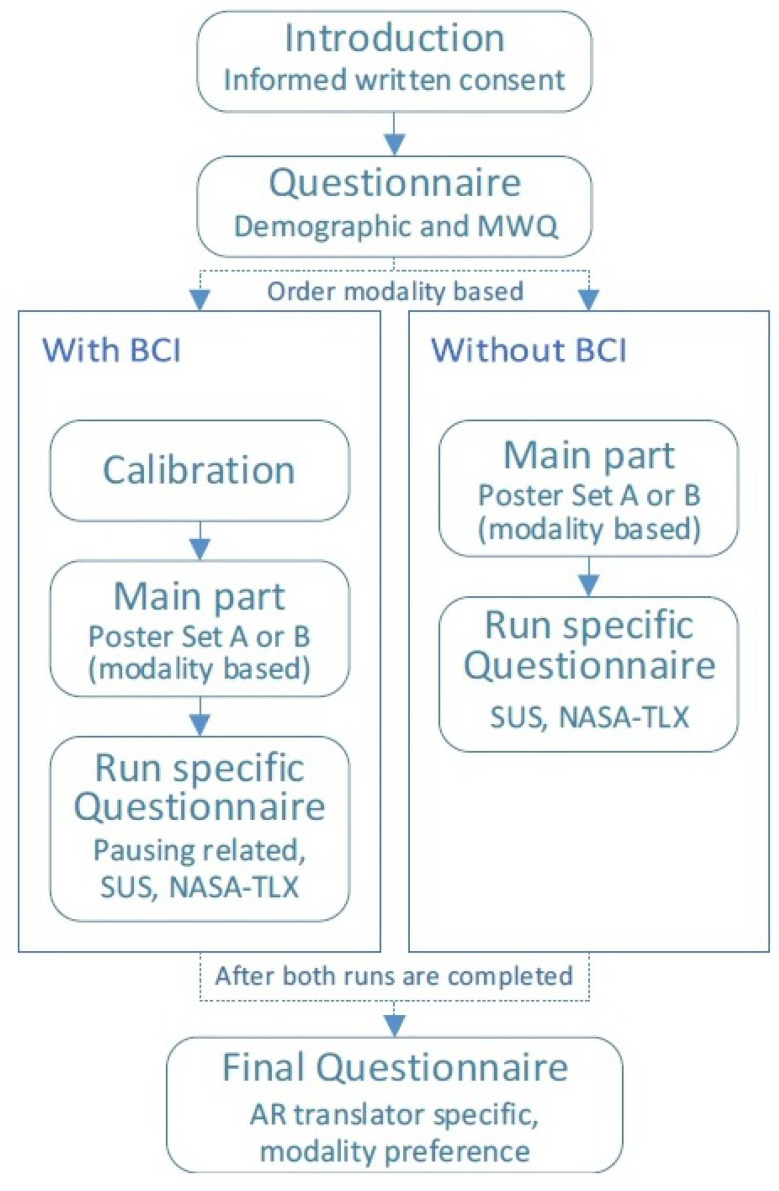
User study procedure overview for the two runs (with and without the BCI to make the system attention-aware).

Figure 4 shows the general structure of the study, which will be explained in the following.

Participants were introduced to the study verbally and through an introductory document, and then provided written consent after being informed about the goal of the study and the data that would be collected. Following that, a demographic questionnaire and the Mind Wandering Questionnaire (MWQ) [47] were completed. The MWQ was collected to find suspiciously high mind-wandering scores because these participants would possibly influence the study results. Afterwards, the app was tested in the participant specific order. Half of the participants began with BCI, while the other half did not. The poster sets were the same; half began with poster set A and half with poster set B, each of which featured four posters. The production and selection of posters will be discussed more below.

For the run with BCI, a calibration of the EEG headset and the training of the classifier were required. The initial calibration was performed by using the built-in impedance measurement of the Muse headset. The training data collection was performed by using four German texts that were read via AR (externally directed attention) and four text-specific questions after each text (internally directed attention). Each of the eight parts lasted roughly 50 s (400 s of training data in total per participant), after which the app informed the participant that enough data had been collected for that part. Participants were instructed not to talk during the training data collection and to refrain from touching the Muse Headset during the remainder of the study, as this could impair the prediction accuracy. The classifier was trained on all the available training data per person.

After the calibration and classifier training, participants were asked to imagine that they are tourists in a foreign country, as that is a fitting use-case scenario for an AR translation app. Virtual posters were displayed on the smartphone screen. To match the overall study narrative during this phase, the content of the texts was related to traveling or about facts of countries. To ensure that participants had something to ponder about within the 50 s, the questions were relevant to the texts and were either open-ended or asked for a lot of information about the text. For example, after reading a paragraph describing Chinese customs, participants were asked to think about both Chinese and other foreign customs.

During the study, the participants’ main task was to read the translations of posters using the AR translation app (externally directed attention) and then answer questions about the content (internally directed attention). The main part structure was communicated verbally and visually to the participants by using a guidance document that included screenshots of each step of the main part. This added an explanation of when and why the application paused in the version with BCI. Participants were informed that pausing may occur during reading and that waiting or attempting to read may cause the pause to be unpaused. The arrangement was identical to the training phase, except that actual posters were pinned to the pinboard and had to be scanned with the camera. Participants were also given the option of rereading the text during the task; however they were instructed to first think about the question and to then rethink their answers completely if they reread the material. Participants were prompted verbally for their replies after each question to reward them and to subjectively judge the difficulty of posters and the quality of answers. The participants were not timed and could spend as much time as they needed to read the poster and complete the exercise.

Participants completed a run-specific questionnaire after each of the two main component runs (with and without BCI). Following the completion of both runs, a final questionnaire was completed.

Participants spend an average of 98 min on the study. To maintain excellent signal quality, the Muse Headset was routinely cleaned before each research.

#### 4.2.3. Questionnaires

Before testing the app, participants filled a demographic questionnaire and the Mind Wandering Questionnaire [47] translated to German. The latter is based on a Likert scale and includes questions to generate a score that shows how likely the person is to start mind wandering. Participants completed a run-specific questionnaire after each run in the main section. The well-known system usability scale (SUS) [48] and the short form of the NASA Task Load Index [49], known as Raw TLX [50], were filled out in this questionnaire (German translations for SUS [51] and Raw TLX [52]). In addition, for the version with BCI, participants answered Likert scalebased questions on translation pauses. These questions were designed to provide answers to the four study hypotheses. Finally, following both runs of the main part, participants completed a questionnaire to rate their overall experience of the AR translator. These questions used a Likert scale and dealt with display smoothness, tracking, visual authenticity, accurate positioning, and content correctness. In the last section of the study, participants chose which version, with or without BCI, they preferred and could provide an open-ended response as to why.

#### 4.2.4. Stimuli

**Figure 5:**
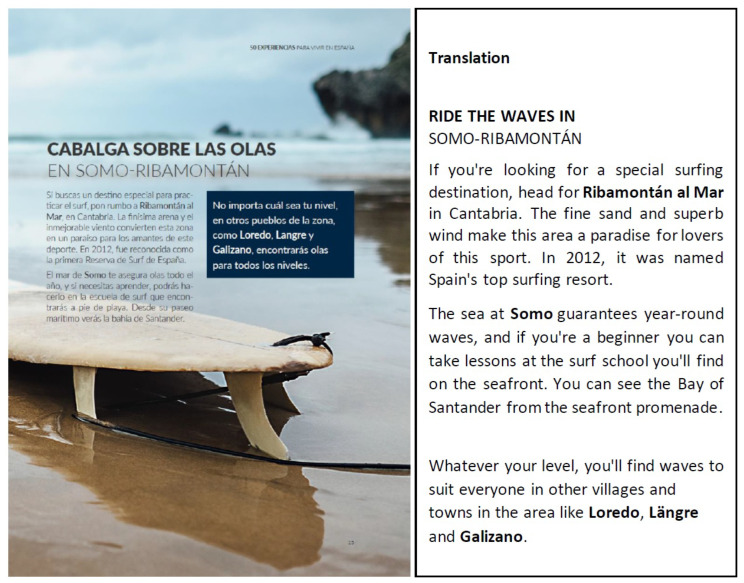
Example poster that was used in the study and the translation (in a separate box for showcase purposes).

An example poster is shown in Figure 5.

The choice of posters for the study plays an important role in testing the mobile BCI for the AR translator, as well as being able to answer *H3* and *H4*. The behavior of the AR translator greatly depends on the appearance and structure of texts. Thus, to sufficiently test the BCI version, a variety of different texts needed to be used to obtain a more holistic review. The creation of the eight posters for the study was done by defining criteria based on what was needed to answer *H3* and *H4*. As *H3* relates to the appearance of texts, answering it also requires a variety of different text appearances similar to what is needed to test the AR translator more holistically.

##### Criteria for the Distractiveness of Translation Updates of Posters

*H3* pertains to the distractiveness of text sticker updates, which depends on the appearance of the text and background on posters. To test if the BCI version of the AR translator may be considered more helpful for text/background combinations that elicit a lot of translation updates, several posters differing in design aspects have to be used. von Mühlenen et al. [53] show that a change in text color strongly captures attention. This effect is likely to be amplified if the background color changes as well. A smaller disparity in text sticker changes is less obvious and consequently less distracting. The visual complexity of a text and its background has a direct relationship to the consistency of text sticker generation. The AR translator replicates a text with an equally colored background more reliably than a text with alternating background colors, because noise and more color shades lead the determined background colors to stray a lot more. This results in a more prominent and attention-grabbing appearance over time, as the switching of text sticker colors between updates is unavoidable.

Another issue is the consistency with which the text stickers are correctly positioned. If the location of text stickers is not consistent between updates, they will shift a lot. Because movement attracts attention [54], consistency in location lowers the distractiveness of updates. A background that is equally colored has fewer potential features for the feature matching procedure. Even if the background lacks good features, the text itself can be used as a reference point. Furthermore, text with poor contrast to the background is unreadable by OCR and even by humans. Hence, it is not possible to create posters with deviating consistency of text sticker placements without significantly compromising legibility and poster authenticity.

The frequency with which text stickers are updated has a direct effect on distractiveness. Changes to the text become more obvious as they occur more frequently, increasing the distractiveness of the previously described distractors. When the text is spread out, less text is in the camera view at the same time, lowering the complexity of the calculations necessary and hence the computation time. As a result, for less dense texts, the update frequency may be higher, increasing distractiveness. Larger typefaces, as well as text layout that spreads out text, are two ways to create lower text density.

Other elements influence distractiveness (change in font size between updates, word ambiguity causing various words to appear between updates), but the ones presented were thought to be the most effective and sufficient to design posters with enough variation.

The criteria are not binary in nature. As a result, in order to make four posters that rise in distractiveness, they must be created in relation to one another. The first poster should be the least distracting, while the last should be the most distracting.

Based on the criteria, four pairs with similar characteristics were created. It was attempted to similarly increase the level of the distraction per difficulty (e.g., gradually increasing font size and spacing between lines to reduce text density from poster 1 to 4).

##### Criteria for the Difficulty of Text and Question Combinations

To evaluate *H4*, the combination of text and question needs to be of varying mental demand. However, this did not need to be as nuanced as the appearance of the posters, as it is only relevant for *H4*. The length of the text is one consideration. The mental demand is often increased when more text provides more information, especially when open-ended questions are asked, where large sections of the text contain part of the answer. Those that require lengthier answers are likely to be more difficult than questions that can be answered in a few words because the participant must recall bigger portions of the text. Two difficulties were created based on the criteria above:

Simple text and question combinations had a word count around 100–150 words. The questions had simple, clearly defined answers (information that is contained in just one or two sentences of the text). The difficult text and question combinations had a word count of around 200–250 words. The questions were open-ended and ask for information contained in large parts of the text. Potentially, the participant needs to reread parts of the text to solve the question, as it is difficult to remember everything needed to solve the question. It should be noted that when using an AR translator, the process of reading is not as straightforward as compared to regular reading. Thus, the general demand is higher than it may seem.

In total, eight posters were used for this study. The posters were split into two groups of four, of which one was used for the configuration with BCI and one for the configuration without BCI (balanced across participants). Within each group, the posters had 4 different levels of difficulty regarding the distractiveness and two levels of difficulty regarding the text and question combinations.

### 4.3. Results

To evaluate both the application and the user study, and to test our hypotheses, we analyzed the classification accuracies and the questionnaire results. For all significance tests, an alpha level of 0.05 was assumed. All correlations were tested by using the Pearson correlation and differences were assessed by using dependent *t*-tests. To increase the readability, all six-digit participant IDs were exchanged by numbers 1–12.

#### 4.3.1. AtAwAR Translate Application Rating

**Figure 6:**
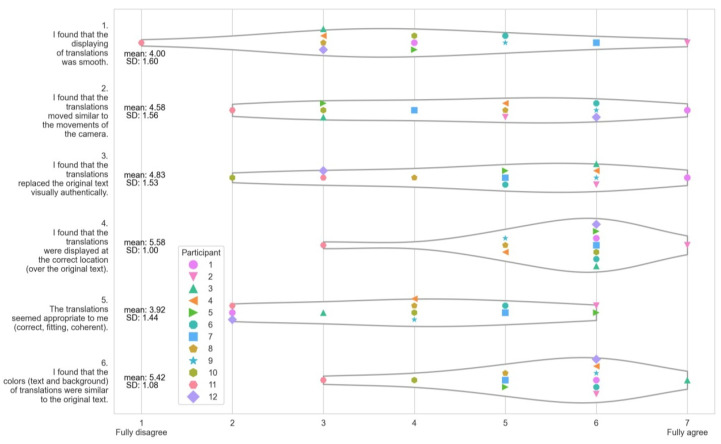
Questionnaire results per participant for the AR translator application rating.

One post-session questionnaire was specifically designed to rate different aspects of the AR Translation application, independent of the BCI aspect. The questions and answers can be seen in Figure 6. For questions 1, 2, 3, and 5, the participants had opposing opinions; however for questions 4 and 6, they agree more. The latter concern the correct arrangement of translations (4) and the matching colors of translations (6), which are likewise the highest-rated aspects (mean 5.6 and 5.4 respectively). The smoothness of translations (1) and the correctness of translations (5) are the lowest-rated features (mean 4.0 and 3.9, respectively). Surprisingly, participants rated “translations matching colors” (6) higher than “translations replaced text visually authentically” (3) (means: 5.4 versus 4.8). When asked directly about the app, many participants stated that the translations were not perfect, making the texts difficult to read in some sections. Nonetheless, the participants were able to understand the core of the texts and, as a result, could respond to questions that frequently covered the most relevant components. Following the experimenter’s subjective judgment, 72% of the answers were rated good (the majority of the questions were answered), 24% okay (about half of the questions were answered), and just 4% poor. In the NASA-TLX, participants responded to the questions “How successful were you in performing the task?” and “How satisfied were you with your performance?” The average result for both modalities in this performance category was 5.5 (SD 2.34), placing them directly in the middle of the scale. Although the majority of the answers were regarded as good, the participants did not appear to agree as much on average. Some participants expressed a desire to be able to interrupt the translations at any time, a function available in Google Translate. The preference of modality had no significant nor interesting correlations with the MWQ.

##### Conclusions

The most criticized aspects of the AtAwAR Translate system were the smoothness and the correctness of translations. The AR translator seems to take too long to display translations. Also, translations were confusing at times, as they are created line by line, lacking coherence between lines. The placement and colors of text stickers were rated the best among the categories, even though some posters were designed to create difficulties regarding color determination for the AR translator. Even though some participants had problems, they were generally able to complete the tasks. Some participants stated that they would not be able to understand anything without the app, but with the AR translator, the Spanish sentences became understandable. We conclude that the quality of the implemented AR translator was good enough to test our hypotheses regarding the mobile BCI integration for attention-awareness.

#### 4.3.2. Classification Accuracies and Pauses

For the BCI version of the AR translator, we evaluated the classification accuracies and analyzed the pauses. These results shine a light on how well the implemented system worked in terms of “attention awareness” and will help explain and discuss the results of the questionnaires.

The accuracy of the attention classification is largely influenced by the calibration and training phase accuracy. On average, the accuracy during the classifier setup was 82.5±8.2% (calculated using a 5-fold cross-validation as described).

**Figure 7:**
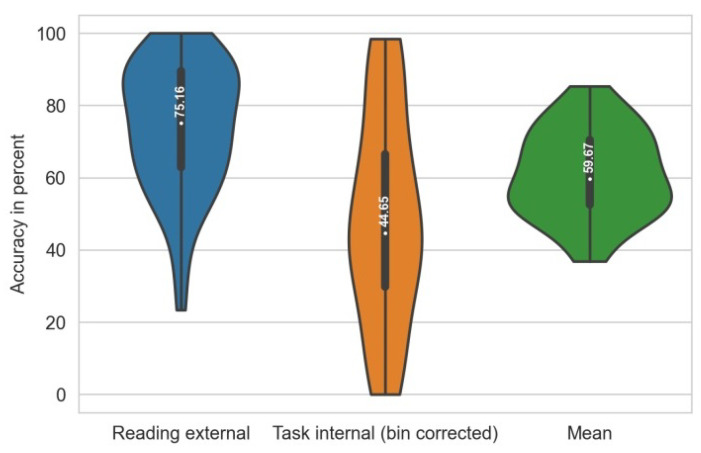
Prediction accuracies for reading (external), task-oriented internal attention and their mean. Indicating the mean, standard deviation and distribution over participants. and mean of both.

As a ground truth, it was assumed that participants’ attention was directed externally in times of reading and internally during question answering. However, because the participants had the option to review the text while answering the question (which would result in externally directed attention within a phase of internally directed attention), we analyzed the reading phases in more detail and found that thinking and rereading resulted in longer task solving times for some trials. Additionally, throughout the course of the question answering phase, the classification accuracy dropped significantly in the middle for some trials. This indicates that the assumed ground truth (internally directed attention) is wrong and needs to be corrected for our offline analysis of the classification accuracy. For trials identified as “thinking and rereading”, phases (each trial was separated into 5 bins) with a high probability for external attention were excluded for this analysis (bin corrected). Figure 7 shows the distribution and mean of classification accuracies per trial during the reading and task separately, as well as the mean accuracy per viewed poster (n = 48 for BCI condition).

The mean classification accuracy in the main part of the study was 59.67% which is comparable to the real-time attention classifier in Vortmann et al. [14]. The classification accuracy in time of external attention (75.16%) was higher compared to the internally directed attention (44.65%). Label noise for this analysis has to be assumed for both trial parts.

**Figure 8:**
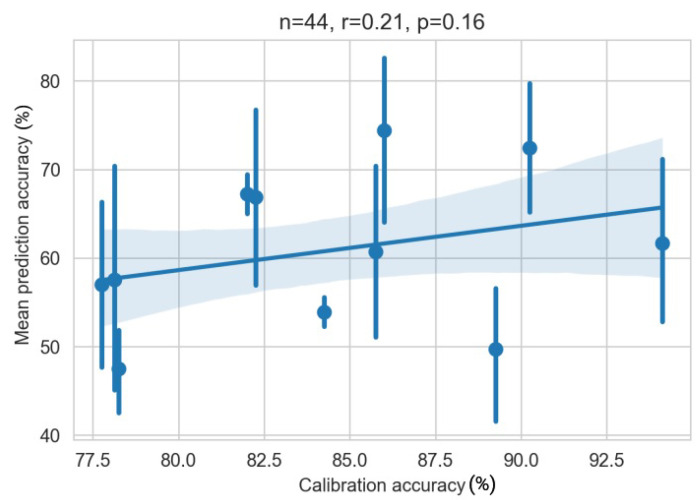
Pearson correlation of calibration and mean prediction accuracy. Each dot with line represents the four posters for one participant. The dot is the mean and the lines are the min and max values of the four posters.

Figure 8 shows the correlation between the classifier calibration accuracy after the training phase and the classification calculated in the offline analysis on the assumed ground truth labels. As p=0.16, the weak positive correlation of r=0.21 is not significant. For most participants, there are also large fluctuations in mean prediction accuracy between the posters.

**Figure 9:**
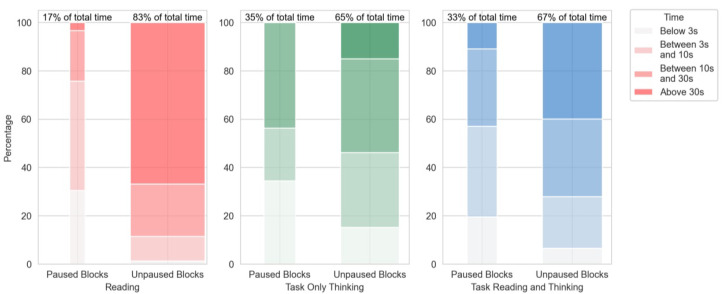
Block during which translations were paused or unpaused separated by reading and task. The task is split into “only thinking” and “thinking and reading”. The data uses the mean of the distributions of participants, so that the length of a run does not increase its weight; each participant had the same weight.

With BCI, participants needed 29 s longer (+23.7%) to read the posters, a statistically significant difference (p<0.01). Surprisingly, this is also true when comparing the modalities of people who favored the BCI (M = 36 s, p<0.01). Participants needed slightly less time with BCI for the task, although this is not statistically significant (p=0.32). In the next step, we analyzed the distribution and length of paused and unpaused translations. Each continuous time interval of either paused or unpaused translations were rated as one block and categorized depending on their length. Figure 9 shows the share of each block length depending on the current trial part. The translations were paused for 17% of the total time participants spend reading the posters. Almost 80% of these pauses were shorter blocks of below 3 s or between 3 and 10 s. The largest part of reading time the translations were not paused for intervals longer than 30 s.

In times of internal attention (only thinking), the translations were paused for 35% of the total time. This was reduced to 33% in the trials there were later identified as thinking and rereading. It can also be noted that the length of the continuous pauses is longer for task parts than for reading parts.

**Figure 10:**
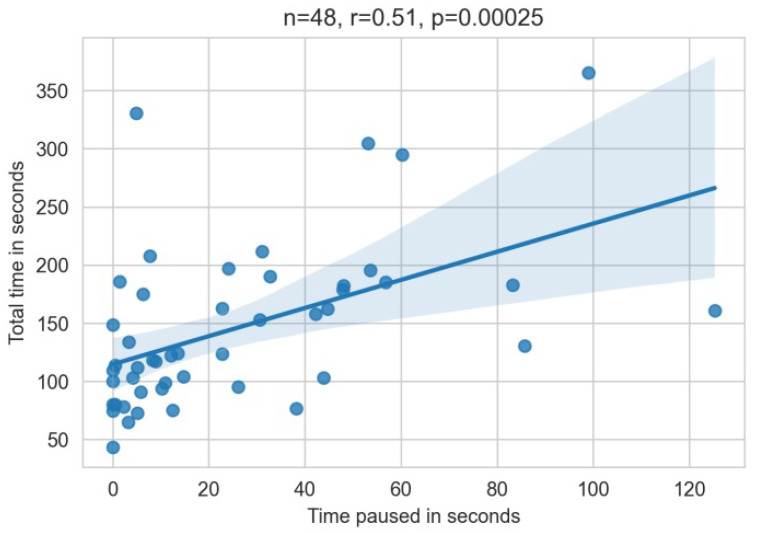
Pearson correlation of the pause time during reading and the total time. Statistics indicated in the figure.

The effect of the translation pauses during the reading part on the total task-solving time was analyzed by using a correlation analysis (see Figure 10). The moderate positive correlation of r=0.51 is highly significant with p<0.001. Thus, longer translation update pauses during reading significantly increased the total task solving times.

##### Conclusions

For the modality with BCI, the attentional classifier reached a median accuracy of around 60%. However, this value is likely affected by label noise, as the study design made a clear separation into thinking and reading difficult. Regarding pauses, the percentage of time paused during reading was around half of that of the task (reading pauses 17%, only thinking task 35% and thinking and reading task 33%). Although a clear difference in pause percentages exists between reading and task, the paused percentage during the task is generally rather low as not even half of the task time was paused. Participants reported to be more affected by the reading pauses.

#### 4.3.3. SUS and NASA-TLX

**Figure 11:**
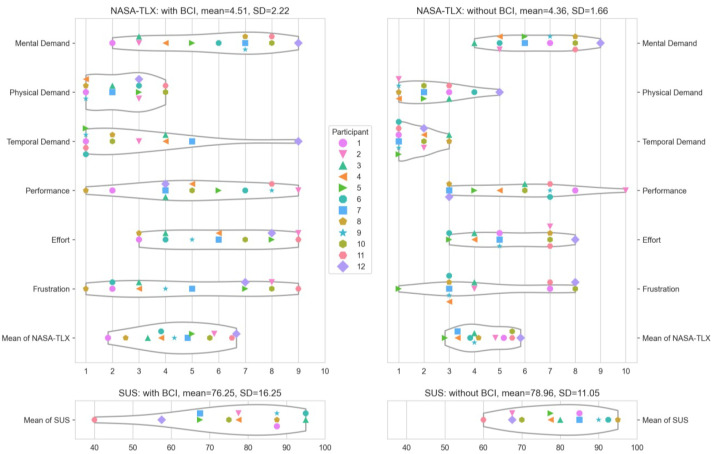
NASA-TLX and SUS ratings per participant for BCI and BCI-less versions of the AR translator app.

The SUS and NASA-TLX questionnaires were provided after each version of the AR translators separately and will be compared to rate the usability of the attention-aware translator compared to a regular AR translator. The version without BCI achieved higher results on the NASA-TLX and the SUS, with a reduced standard deviation, for both questionnaires. The higher SD for the version with BCI could be attributable to the fact that some people found the pause useful, while others found it annoying, resulting in polarization. The BCI version of the translator achieved an average SUS score of 76, and the BCI-less version achieved a score of 79. According to Bangor et al. [55], these values fall between between “good” (71.4) and “great” (85.5). The version without BCI is slightly closer to “excellent,” whereas the version with BCI is slightly closer to “good.” The answers per person for both questionnaires can be seen in Figure 11.

To evaluate the differences between the NASA-TLX categories for both versions of the system, paired t-tests were performed, but no significant differences were found. The biggest rated differences were present for the mental and temporal demand (see Table 2). The overall NASA-TLX and SUS score ratings were also not significantly different.

##### Conclusions

The SUS and NASA-TLX scores were better for the version with BCI. The biggest difference for the NASA-TLX was the temporal demand, which might be due to the fact that participants required on average 29 s longer to read posters with the BCI (p<0.01). The SUS score with BCI is 79 and without 76, both can be considered “good” according to Bangor et al. [55].

#### 4.3.4. Hypotheses

**Figure 12:**
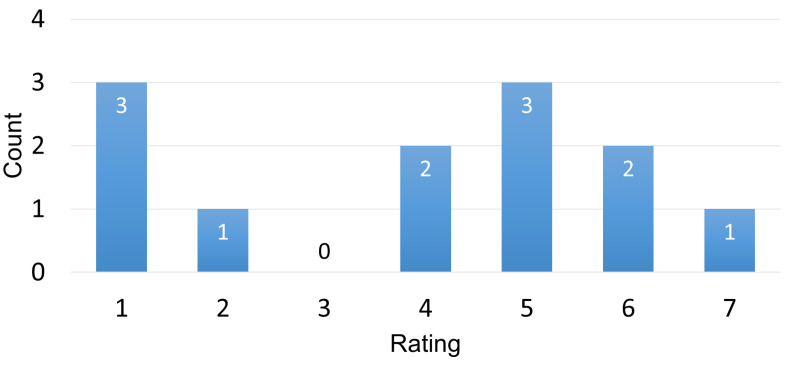
Answers to the statement “I found the automatic pausing to be more distracting than helpful”. 1 = fully disagree, 7 = fully agree; n = 12.

The main aspect of this study was to find out whether an attention-aware AR translator would be superior to an attention-unaware system. When asked which modality they preferred, 58.3 percent chose the version without BCI and 41.7 percent liked the version with BCI. The major reasons for preferring an unaware version were that pausing interfered with the reading and the Muse headset was distracting. Those who favored the attention-aware version said the text was more legible since it did not update as frequently, and it seemed less stressful because the app adapted to their cognitive state. Figure 12 shows the participants agreement with the statement “I found the automatic pausing to be more distracting than helpful”. Participants who liked the BCI version rated the question with an average of 2.8, whereas the other group gave it a rating of 4.7. The latter is closer to a neutral value of 4. The participants who liked BCI leaned more towards 1 than the other group did towards 7, indicating a clearer preference.

**Figure 13:**
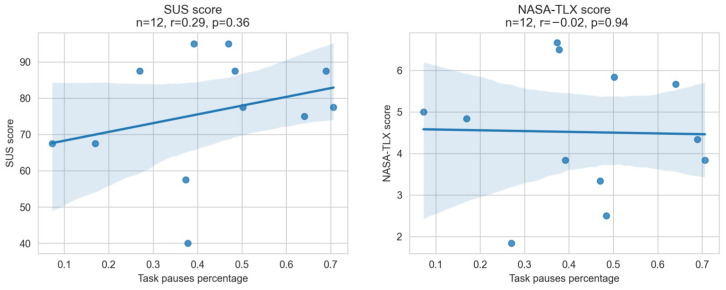
Questionnaire scores of SUS and NASA-TLX correlated (Pearson) with the percentage of pauses during task. For the task, the data of OT and the first bin of TR was used; as for these it is more likely that participants were thinking.

*H1* suggested that ”The larger the percentage of pauses during thinking, the higher the experienced usability and the lower the task load.“ To evaluate the hypothesis, we calculated the correlation of the SUS and NASA-TLX scores with the task pauses percentage (see Figure 13).

Regarding the task load (represented by the NASA-TLX), there was no correlation between the scores and the percentage of pauses during the task. For the usability assessment (represented by the SUS), we found a weak positive correlation of r=0.29 which is, however, not significant (p=0.36). We would have expected a positive correlation between pauses and the SUS score and a negative correlation between the pauses and the NASA-TLX. With the current results, *H1* can not be supported but the SUS results are promising for a larger test set.

**Figure 14:**
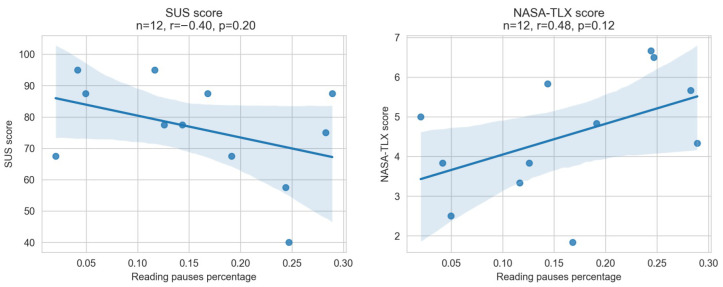
SUS and NASA-TLX correlated (Pearson) with the percentage of time that translations were paused during reading. The pauses percentage is the mean of the four posters.

*H2* stated “The larger the percentage of pauses during reading, the lower the experienced usability and the higher the task load.” To evaluate this hypothesis, we calculated the correlation of the SUS and the NASA-TLX scores with the translation pasues during reading (see Figure 14).

Although both correlations are not statistically significant with p-values of 0.2 and 0.12, they do demonstrate a tendency that supports *H2* with absolute r-values greater than 0.4. The bigger the percentage of pauses, the worse the score for both questions (a higher score for NASA-TLX is worse). As a result, it is likely that *H2* is proven to be true with more participants. At this point, the results do not support *H2*.

**Figure 15:**
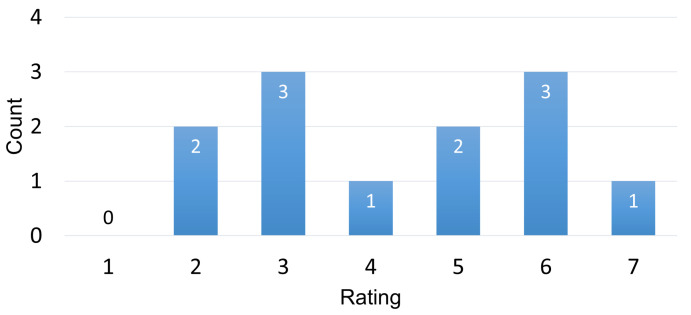
Answers for agreement to the statement related to *H3* “The more distracting translations were during thinking, the more helpful the pausing was for me”. 1 = fully disagree, 7 = fully agree; n = 12.

For the evaluation of *H3*, participants were asked how much they agreed with the statement “The more distracting translations were during thinking, the more helpful the pausing was for me,” which they would agree with if H3 were accurate. The results are shown in Figure 15.

The average score was 4.3 (SD 1.7), indicating that participants tended to agree more than disagree. This supports *H3*. Participants were also asked if they thought the pausing was more distracting than useful (see Figure 12), and those who thought it was more distracting tended to disagree with the statement in Figure 15. The Spearman’s correlation yielded an r-value of −0.78 with a *p*-value of 0.01, indicating that participants appeared to agree with this statement if they regarded the pausing to be useful.

**Figure 16:**
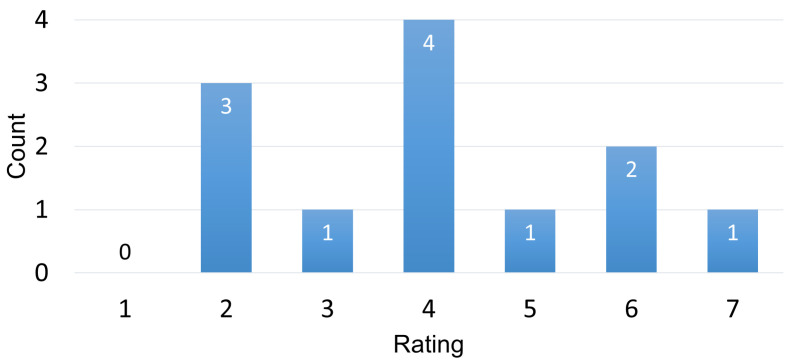
Answers for agreement to the statement related to *H4* “The more I had to think during a question, the more helpful the pausing was for me”. 1 = fully disagree, 7 = fully agree; n = 12.

Finally, *H4* was evaluated by using another questionnaire question. *H4* stated that “The more demanding the combination of text and question is, the more helpful pauses are during thinking.” The agreement to the statement “The more I had to think during a question, the more helpful the pausing was for me” can be seen in Figure 16.

The mean of 4.1 (SD 1.7) is lower than for the *H3* statement. The results for this statement were also correlated with the results for the statement of whether participants felt the pausing to be more distracting than useful, and a negative correlation (r=−0.63, p<0.05) was found.

##### Conclusions

There is evidence for both *H1* and *H2*, because task pauses correlate with high SUS and NASA-TLX scores and reading pauses with low SUS and NASA-TLX scores, albeit this was not statistically significant. Although *H3* and *H4* are likewise difficult to answer, participants who preferred the version with BCI appeared to agree with the hypotheses’ statements.

## 5. Discussion

The case study to test the implemented AtAwAR Translate system revealed interesting insights into user preferences regarding the setup and application behavior, as well as the use case itself.

### 5.1. Use Case

As an authentic mobile BCI setting use case, we decided to use an AR translator because it is frequently used “in the wild”. By using a smartphone to display the AR content is automatically less visually distracting than a head-mounted display that is typically used for augmented reality. The usability of such AR glasses could probably be improved even more by adding attention awareness compared to the cell phone application.

The content for the translations was made up of posters containing travel-related information. As previously stated, only contiguous texts were employed in the study; nevertheless, discontiguous texts may also be translated by users. Moreover, these texts are likely to have a distinct distribution of reading and thinking time, because disjoint texts require less reading (e.g., book covers, shop window labeling, menus, consumer product labels, maps) and thinking is significantly dependent on the user. It is conceivable that a user studies a menu and spends considerable time inwardly discussing which food to order. In such an instance, the AR translator would have more chances to halt when thinking than while reading. Such a use case might be even more appropriate to test the enhancement through attention-sensitive BCIs.

### 5.2. AtAwAR Translate Application

Participants were generally able to use the translator to understand texts of foreign language, judging by the subjective rating of their answers to the questions (72% of answers considered good). The majority of participants preferred the version without BCI (58.3%, n = 7). The main reason for the preference of modality was the pausing during reading. For some participants, the reading pauses made the text more legible, as the text did not switch as much. Other participants disliked the pauses, as they interrupted the reading flow. The pausing during thinking did not play a deciding role; however it seems that it was generally regarded as positive. Because of the study design, the appearance of text stickers played only a minor role, which is presumably why the main complaints participants indicated in the AR translator questionnaire were the smoothness of translations and the quality of translations, both of which relate to understanding. Needless to say, the legibility of text stickers was critical for understanding in the study, although it did not really matter if a text sticker had red or black text to understand it. It is interesting that participants evaluated “matching colors of translations” higher than “translations replaced text visually authentically” (means: 5.4 versus 4.8). The purpose of an AR translator is to create visually accurate text stickers, and matching text stickers merely in color does not appear to be sufficient. It should be emphasized that half of the posters were intended to cause problems for the AR translator. Posters 3 and 4 of both poster sets have letters with low contrast to the backdrop, whereas the background is an image with many varied colors that alternate often throughout the image. This is particularly difficult for OCR, and likely negatively affected the ratings of participants regarding the answers related to visual effects. Another issue raised by participants was the quality of translations. Because the OCR recognized text line by line, the translations were done line by line. It would make sense to utilize an OCR that returns text paragraph by paragraph, as the context between lines is critical for translation tools to construct meaningful translations; however, PaddleOCR does not support this. Grouping lines after OCR is a non-optimal workaround, as paragraph detection within OCR is the faster method. This is because the image is already being processed by the OCR, making it more resource-efficient.

Overall, the visual authenticity of text stickers was rated well, but there is still room for improvement of the AR translator application in general. This is most obvious if it is used to understand relatively large texts. However, the quality of the implemented application was good enough for our purposes and the mentioned shortcomings most likely did not severely interfere with the study outcome.

### 5.3. BCI Integration

The general setup using two smartphones and the Muse headset worked very well, and calibration times were fast and easy. The system was rather comfortable to wear and the data quality seems sufficient for these purposes. The achieved classification accuracy of almost 60% was comparable to other real-time BCI applications in lab settings [6]. The main issue in this study was the label noise due to the rereading of text in time of internally directed attention. For future studies, it should be assured that externally and internally directed attention are clearly separable for the generation of ground truths to evaluate the classifier. Another study design-related issue is the learning effect that may cause the second modality in execution order to be rated better. That is why looking at individual participants may not be expedient. Furthermore, judging upon the subjective answer quality, the difficulty of the two poster sets appears to differ slightly.

It is possible to improve the accuracy by adjusting the calibration configuration. Participants used augmented reality to read German literature. Although it is encouraging that the text was read with AR in a manner similar to that of the AR translator, there is likely still a difference in elicited brain signals when comparing the calibration reading to the reading with the AR translator. Reading with an AR translator differs from conventional reading in that the flow of reading is frequently disrupted, either because translations swap while reading or because they do not always make sense. This is likely to result in confusion, possibly even frustration, or other reactions. A more specific calibration, in which participants read using the AR translator, would most certainly improve overall accuracy. Similarly, the thinking phase of the calibration could entail needing to glance at the text while the AR translator is paused. The calibration and main section of the study would then be more comparable.

In the long run, however, it would be desirable to exclude the need for collecting person-dependent training data to set up the classifier. This would increase the usability of a mobile BCI smartphone system by a lot. Vortmann and Putze [44] addressed the idea of person-independent EEG-based classification for internally and externally directed attention and found that neural networks outperform an LDA approach for 4-s data windows in such cases. The analysis in their paper were performed offline and a lot of training data was available for the classifier. No information about the computation time is given. For a training-free real-time application the choice of classification algorithm would have to be reevaluated. Again, the goal would be to find a compromise between computation time and classification accuracy.

### 5.4. Attentional State Application Adaptation

The attention-aware AR translator in this study was designed to update the translations while the participants were reading the text to adapt to deliberate camera movements, and it was supposed to pause the updates during times of perceptual suppression and internally focused thoughts to avoid goal-oriented thought disruption. The comparison of both versions of the system showed that participants decided for or against a version based on the pausing during reading (which was due to wrong attentional state classification results). Although 4 participants expressed that the pausing interrupted their flow of reading, another 4 participants expressed that this initially unwanted system behavior actually helped them to focus on the reading. Only one participant commented on the intended pauses while thinking, stating that the app met their needs. Another participant claimed that pausing while thinking was beneficial, but because they were focused on the work, it had no effect on them. This opinion was echoed by several subjects following the study when they were interviewed.

It was not anticipated while preparing the questionnaire or planning the study that participants would find the pause while reading useful. As a result, some questions did not directly inquire about pausing when thinking, but rather about halting in general. As a result, it is unclear whether participants felt the pausing to be beneficial because of the reading or because of the thinking, and by how much. According to the open-ended responses of those who preferred the version with BCI, pausing during reading was more useful than pausing during thinking. It is unknown how much of their choice is dependent on the pauses during thought. When asked about the pauses during thinking, the participants’ responses were typically positive. However, because no participant explicitly said this in their open-ended response in the final questionnaire, those pauses do not appear to be a determining factor.

The way the classification result was integrated into the behavior of the application sometimes led to pauses of over 30 s before a new update would appear. This is far too long and should be restricted in later experiments.

## 6. Conclusions

We performed a user study to test the effects of the BCI integration into an AR translator application. The main novelty and contribution of this research was the mobile setup consisting of a smartphone and a consumer-grade EEG headset in our AtAwAR Translate system. We hypothesized that attentional state adaptation of text updates reduces distraction caused by the AR elements and increases the usability of the system. These hypotheses could not yet be proven with the current data. However, there are several other inferences and results, as well as indications that attention sensitivity can be helpful. This was the first attempt at developing a mobile BCI system using a smartphone and a low-cost EEG headset with few electrodes to add attention awareness to an AR application. Previously, the idea that the application’s attention-awareness would improve an AR application was only tested for head-mounted displays and in a laboratory setting. We chose an AR translator inspired by Google Translate as a use case because it is a popular AR smartphone app that is frequently used on the road and provides several switches between internally and externally directed attention. A travel scenario was used for the user study, and foreign tests had to be read and understood.

The findings of our user study did not entirely support the claim that the BCI improves usability. The main limitations of the suggested framework are the accuracy of the classifier and the user-specific preferences of the adaptation behavior. However, the achieved performance and ease of setup demonstrated that the system design is promising. Other use cases, usage scenarios, display devices, or applications should be investigated for future studies aimed at mobile attention-aware AR systems. For example, the precise nature of the interface’s attention adaptation or system behavior must be thought through and efficient in order to reduce distractiveness.

## Figures and Tables

**Table 1:** All apps found in the Google Play Store (Android) with the query “AR translate” and “Camera translate” on 26 February 2021. Many more translation apps are available that do not feature AR, so only the top ones were analyzed for this comparison. Langs., languages; RT, real time.

						Text Replace	
App Name	Author	Release	Downloads	Langs.	RT	AR	Text Display
Google Translate AR	Google	2015	500M+	109	yes	yes	Replacement
Microsoft Translator	Microsoft	2015	50M+	22		(yes)	Overlaid
Camera Translator	EVOLLY.APP	2017	10M+	56		(yes)	Overlaid
Camera Translator	Fox Solution	2018	1M+	163			Separate
Translator for Texts, Websites & Photos	Octaviassil	2018	1M+	108			Separate
Cam Translate	Xiaoling App	2019	100k+	28			Separate
Language Translator	Touchpedia	2021	50k+	105			Separate

**Table 2:** Mean and standard deviation of the NASA-TLX categories.

NASA-TLX Category	With BCI		Without BCI		Difference
	M	SD	M	SD	
Mental Demand	5.75	2.3	6.5	1.57	−0.75
Physical Demand	2.33	1.15	2.33	1.3	0
Temporal Demand	2.83	2.4	1.67	0.78	1.17
Performance	5.25	2.45	5.75	2.22	−0.5
Effort	6	2.22	5.41	1.72	0.58
Frustration	4.92	2.78	4.5	2.35	0.41

## Data Availability

The data and application implementation presented in this study are openly available at https://osf.io/bxdkw/.
